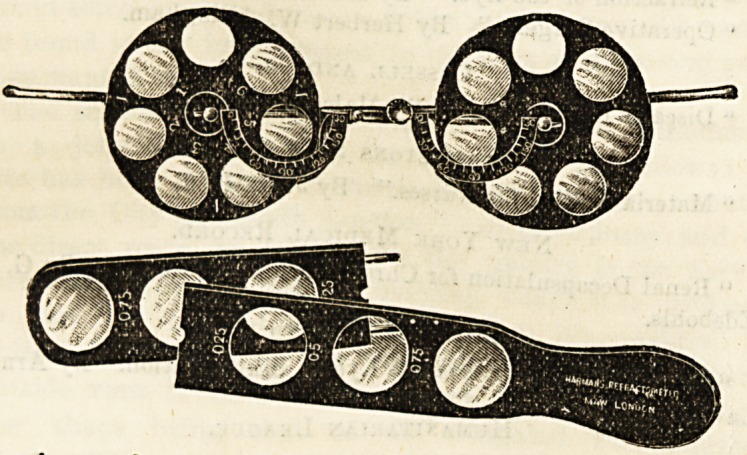# New Appliances and Things Medical

**Published:** 1903-04-18

**Authors:** 


### NEW APPLIANCES AND THINGS MEDICAL.

**[We shall be glad to receive at our Office, 28 & 29 Southampton Street, Strand, London, W.C., from the manufacturers, specimens of all new preparations and appliances which may be brought out from time to time.]**

### NEW REFRACTOMETER.

(Messrs. S. Maw, Son, and Sons, 7–12 Aldersgate Street, London, E.C.

The principle on which this refractometer acts is simple and such as can be utilised by practitioners and others who possess neither the ordinary expensive test cases nor the experience of a practised ophthalmic surgeon. The apparatus consists of a face-piece, on which rotate two fenestrated discs, the one containing six (+) lenses, the other containing the same number of (−) lenses. By rotating the discs the lenses come successively before the eyes. If a higher sphere, or one containing fractions of a dioptre is required, a fraction-rule, which is supplied with the instrument, is held vertically before the eye in front of the fixed lens. The dependent frames for the reception of additional lenses are fixed in such a manner that on reversing the instrument each automatically retains the pendent position. The frame is adjustable for binocular inter-pupillary distance by a sliding-bar bridge and thumb screw. Astigmatism may also be readily determined. We regard this new refractometer as a very practical and useful instrument for the use of practitioners for the ordinary purposes of sight-testing.

### DR. HÖGYES' HYGIENIC ASBESTOS SOLES.

The above asbestos soles are intended for the purpose of preventing the various forms of sore feet to which those who have to do much walking and much standing are exposed. It is claimed for these soles that they prevent the feet slipping about within the boot, thus avoiding the friction which leads to corns, bunions, and inflammation. No doubt the roughened surface of the asbestos material will accomplish this object. Asbestos is an efficient protection against extremes of hot and cold, and by thus shielding the feet from sudden changes of temperature, as well as from damp, it must act efficaciously in preventing the various rheumatic affections to which the feet are liable.

## Figures and Tables

**Figure f1:**